# Efficacy of prophylactic antibiotics for the prevention of neutropenic fever in patients with multiple myeloma receiving high-dose cyclophosphamide for stem cell mobilization

**DOI:** 10.1007/s00277-023-05537-3

**Published:** 2024-01-25

**Authors:** Li-qiong Hou, Jun-Ru Liu, Jing-Li Gu, Mei-Lan Chen, Li-Fen Kuang, Bei-Hui Huang, Wai-yi Zou, Juan Li

**Affiliations:** Department of Hematology, The First Affiliated Hospital of Sun Yat-Sen University, 58 Second Zhongshan Road, Guangzhou, 510080 China

**Keywords:** Neutropenic fever, Mobilization, Antibiotic prophylaxis, Myeloma

## Abstract

High-dose cyclophosphamide (HD-Cy) (3 g/m^2^) plus granulocyte colony-stimulating factor (G-CSF) is a very effective regimen for peripheral blood stem cell (PBSC) mobilization. Unfortunately, it is associated with an increased risk of neutropenic fever (NF). We analyzed the effect of NF on PBSC apheresis results and the efficacy of prophylactic antibiotics for the prevention of NF associated with HD-Cy plus G-CSF for PBSC mobilization in patients with newly diagnosed multiple myeloma (MM). First, patients were divided into NF ( +) and NF ( −) groups according to whether they suffered from NF during mobilization. Second, we divided patients into an antibiotic prophylaxis group and a nonantibiotic prophylaxis group according to whether antibiotic prophylaxis was used during the mobilization period. Our study showed that NF( +) patients (*n* = 44) had lower CD34 + cell dose collection (median 2.60 versus 5.34 × 10^6^/kg, *P* < 0.001) and slower neutrophil engraftment and platelet engraftment (median 11 versus 10 days, *P* = 0.002, and median 13 versus 11 days, *P* = 0.043, respectively) than NF( −) patients (*n* = 234). Of note, the nonantibiotic prophylaxis group patients (*n* = 30) had a 26.7% incidence of NF. In the patients receiving antibiotic prophylaxis (*n* = 227), the incidence was reduced to 9.3% (*P* = 0.01). The antibiotic prophylaxis patients had higher CD34 + cell collection (median 5.41 versus 2.27 × 10^6^/kg, *P* < 0.001) and lower hospitalization cost of mobilization ($ median 3108.02 versus 3702.39, *p* = 0.012). Thus, our results demonstrate that NF is associated with lower CD34 + cell collection and that antibiotic prophylaxis can reduce the incidence of NF and improve stem cell mobilization and collection outcomes, which reduces the hospitalization cost of mobilization.

## Introduction

In an era of novel therapies, autologous stem cell transplantation (ASCT) remains an important treatment to improve the long-term survival of patients with multiple myeloma (MM). Peripheral blood stem cell (PBSC) mobilization and collection of adequate numbers of CD34 + cells are necessary to proceed with ASCT. The combination of high-dose cyclophosphamide (HD-Cy) (3–5 g/m^2^) plus granulocyte colony-stimulating factor (G-CSF) has been considered the standard mobilization regimen for MM. It has high efficacy collection yield and low mobilization failure rates [1, 2]. However, neutropenic fever (NF) is common. The incidence of NF in patients who received HD-Cy plus G-CSF without antibiotic prophylaxis for PBSC mobilization varies in the literature from 25 to 73.8% [3–6]. NF may lead to mobilization failure and missed opportunities for stem cell collection. The costs of mobilization failure and second mobilization attempts are considerable. After noting a high incidence of NF, many centers began using antibiotics to prevent infection during PBSC mobilization [7–9]. In 2006, our center began to utilize HD-Cy plus G-CSF for PBSC mobilization, and we also noted a high rate of NF in this group. Our center also began giving antibiotics to prevent infection during stem cell mobilization in 2010.

However, the risk factors for NF or the impact of NF on PBSC mobilization and collection associated with HD-Cy plus G-CSF for stem cell mobilization is still unclear. At present, only a few studies discuss the effect of NF on PBSC mobilization and collection, and the results are contradictory [10, 11]. In addition, although many centers began using antibiotics to prevent infection during PBSC mobilization, the efficacy and safety of prophylactic use of antibiotics in mobilization remain unclear. There were no phase II or randomized studies that have evaluated the safety and efficacy of prophylactic antibiotics among patients receiving HD-Cy plus G-CSF mobilization. Only two retrospective studies have analyzed the effects of prophylactic antibiotics on the incidence of NF and on the mobilization and collection results, although the results were inconsistent [12, 13]. Therefore, we performed a retrospective analysis on patients with MM who underwent mobilization with HD-Cy plus G-CSF to identify the impact of NF on apheresis outcomes and the efficacy and safety of prophylactic antibiotics for the prevention of NF and stem cell mobilization and collection.

## Methods

### Mobilization strategies and antimicrobial regimen

All 278 patients with newly diagnosed MM undergoing ASCT with HD-Cy (3 g/m^2^) plus G-CSF mobilization between June 2006 and December 2021 were enrolled in this study at the First Affiliated Hospital of Sun Yat-sen University. All patients were mobilized for the first time. Patients mobilized with HD-Cy plus G-CSF received Cy 3 g/m^2^, and on the next day, G-CSF 300 μg/d was administered, which was continued until collection was complete. If a patient received PEGylated recombinant human granulocyte colony-stimulating factor (PEG G-CSF), one dose of PEG G-CSF 6 mg was administered 24 h after Cy. All patients developed neutropenia, and PBPC collection began when the peripheral WBC count reached more than 2 × 10^9^/L. The primary collection goal according to our institutional protocol was 2 × 10^6^ CD34 + cells/kg, with an additional 2 × 10^6^ CD34 + cells/kg preferred for a backup. Between June 2006 and April 2010, 30 patients underwent HD-Cy plus G-CSF mobilization without a specific antimicrobial prophylaxis program. Between May 2010 and December 2021, patients received antimicrobial prophylaxis as follows: imipenem/cilastatin 0.5 g i.v. Q8H. This was started on the first day with a neutrophil count of 0.5 × 10^9^/L and continued until the patient was no longer neutropenic. We excluded patients who were already infected and required antibiotic treatment before the onset of a neutrophil count of 0.5 × 10^9^/L after mobilization.

### Outcome measures

The major endpoints were CD34 + yield and the incidence of febrile episodes. Neutropenic fever was defined as a temperature ≥ 38.3 °C or ≥ 38 °C for two episodes more than 1 h apart, and an absolute neutrophil count (ANC) < 0.5 × 10^9^ cells/L or < 1 × 10^9^ cells/L, expected to decrease below 0.5 × 10^9^ cells/L within 48 h [14]. Secondary endpoints of our study were day 1 CD34 + cell yield, the number of apheresis times required for the collection of CD34 + cells greater than 2 × 10^6^/kg, the proportion of patients who achieved > 2 × 10^6^/kg CD34 + cells, the proportion of patients who achieved > 4 × 10^6^/kg CD34 + cells, the proportion of patients who achieved > 5 × 10^6^/kg CD34 + cells, the time from mobilization to apheresis, the number of days in hospital from the beginning of mobilization to the end of the collection, transplant outcomes such as time to neutrophil and platelet engraftment (defined as neutrophil > 0.5 × 10^9^ on three separate measurement or platelet > 20 × 10^9^ sustained on three separate measurement), progression-free survival (PFS), and overall survival (OS). The proportion of patients obtained a collection target greater than 2 × 10^6^ CD34 + cells/kg, the infection of multidrug-resistant bacteria and invasive fungal infection, and the hospitalization cost of mobilization and apheresis.

### Statistical analysis

SPSS 23.0 was utilized for statistical analysis. Patient characteristics and stem cell mobilization and collection results were compared between two groups using either the chi-square test or Wilcoxon rank sum test. Logistic regression analysis was used to identify univariable risk factors for NF. Stepwise logistic regression analysis with a variable entry criterion of *P* ≤ 0.10 and a variable retention criterion of *P* ≤ 0.05 was used to identify multivariable risk factors for NF. The results are summarized as the odds ratio (OR) and 95% confidence interval (CI). Progression-free survival (PFS) was calculated from the start of mobilization to disease progression, death, or the last follow-up, and overall survival (OS) was calculated from the start of mobilization until death or the last follow-up. The Kaplan–Meier method was performed for survival analysis, and the differences were analyzed using the log-rank test. Univariate and multivariate analyses of features predicting survival were examined using the Cox proportional hazards model. Stepwise Cox proportional hazards analysis with a variable entry criterion of *p* ≤ 0.10, and a variable retention criterion of *p* ≤ 0.05 was used to identify multivariable risk factors. The results are summarized as the hazard ratio (HR) and 95% CI.

## Results

### Patient demographics of the NF (+) group and NF (−) group

A total of 278 patients were included. NF was seen in a total of 44 patients. Table 1 shows that the NF( +) group and NF( −) group did not differ significantly in terms of age, sex, ISS stage, number of prior chemotherapy regimens, disease status before mobilization, and prior exposure to lenalidomide or alkylating agents. Only two patients in the NF (-) group received radiation therapy before mobilization (Table 1).

**Table 1:** Baseline characteristics at diagnosis of study population of NF( +) group and NF( −) group

Variable	NF ( +) (*n* = 44)	NF ( −) (*n* = 234)	*P* value
Mean age, year (± SD)	54 ± 9.42	51.86 ± 9.10	0.760
Male, *n* (%)	26 (59.09%)	142 (60.68%)	0.485
Time from diagnosis to mobilization (mo), median (IQR)	5.0 (4.00,6.00)	5.1 (4.58, 6.77)	0.840
ISS, *n* (%)			0.060
I	10(22.72%)	79(33.76%)	
II	18(40.91%)	105(44.87%)	
III	16(36.36%)	48(20.51%)	
Prior radiation therapy	0	2(0.85%)	1.000
Number of prior chemotherapy regimens, *n* (%)			0.896
1	35 (79.55%)	183 (78.21%)	
2	7 (15.91%)	42 (17.95%)	
≥ 3	2 (4.54%)	9 (3.85%)	
Disease status before mobilization, *n* (%)			0.446
CR	12 (27.27%)	86 (36.75%)	
VGPR	22 (50.00%)	108 (46.15%)	
PR	8 (18.18%)	35 (14.96%)	
SD	2 (4.55%)	4 (1.71%)	
PD	0	1 (0.43%)	
Previous lenalidomide exposure, *n* (%) ≥ 4 cycles	5 (11.36%)	15 (6.41%)	0.334
Prior exposure to alkylating agent, *n* (%)	3 (6.87%)	13 (5.56%)	0.725
Plasma cell in the bone marrow (%) median (IQR)	22% (10.01%, 38.13%)	24% (12.10%, 41.32%)	0.465
Hemoglobin at diagnosis (g/L), mean ± SD	94.52 ± 18.61	99.83 ± 24.66	0.105
Serum creatinine at diagnosis (umol/L), median (IQR)	90 (70.00, 144.75)	82 (63.13, 111.75)	0.173
Serum albumin at diagnosis (g/L), mean ± SD	34 ± 8.01	35 ± 7.23	0.577
Serum calcium at diagnosis (mmol/L), median (IQR)	2.41 (2.24, 2.68)	2.36 (2.21, 2.49)	0.097
LDH at diagnosis (U/L), median (IQR)	157 (124.31, 227.00)	167 (130.13, 203.41)	0.916
Type of myeloma, *n* (%)			0.278
IgG	28 (63.64%)	119 (50.85%)	
IgA	9 (20.45%)	54 (23.09%)	
IgD	1 (2.27%)	9 (3.85%)	
Light chains only	6 (13.64%)	52 (22.22%)	

*ISS* international staging system, *CR* complete response, *VGPR* very good partial response, *PR* partial response, *SD* stable disease, *PD* progressive disease, *LDH* lactate dehydrogenase

### Stem cell mobilization and transplant outcomes of NF (+) group and NF(−) group

**Fig. 1:**
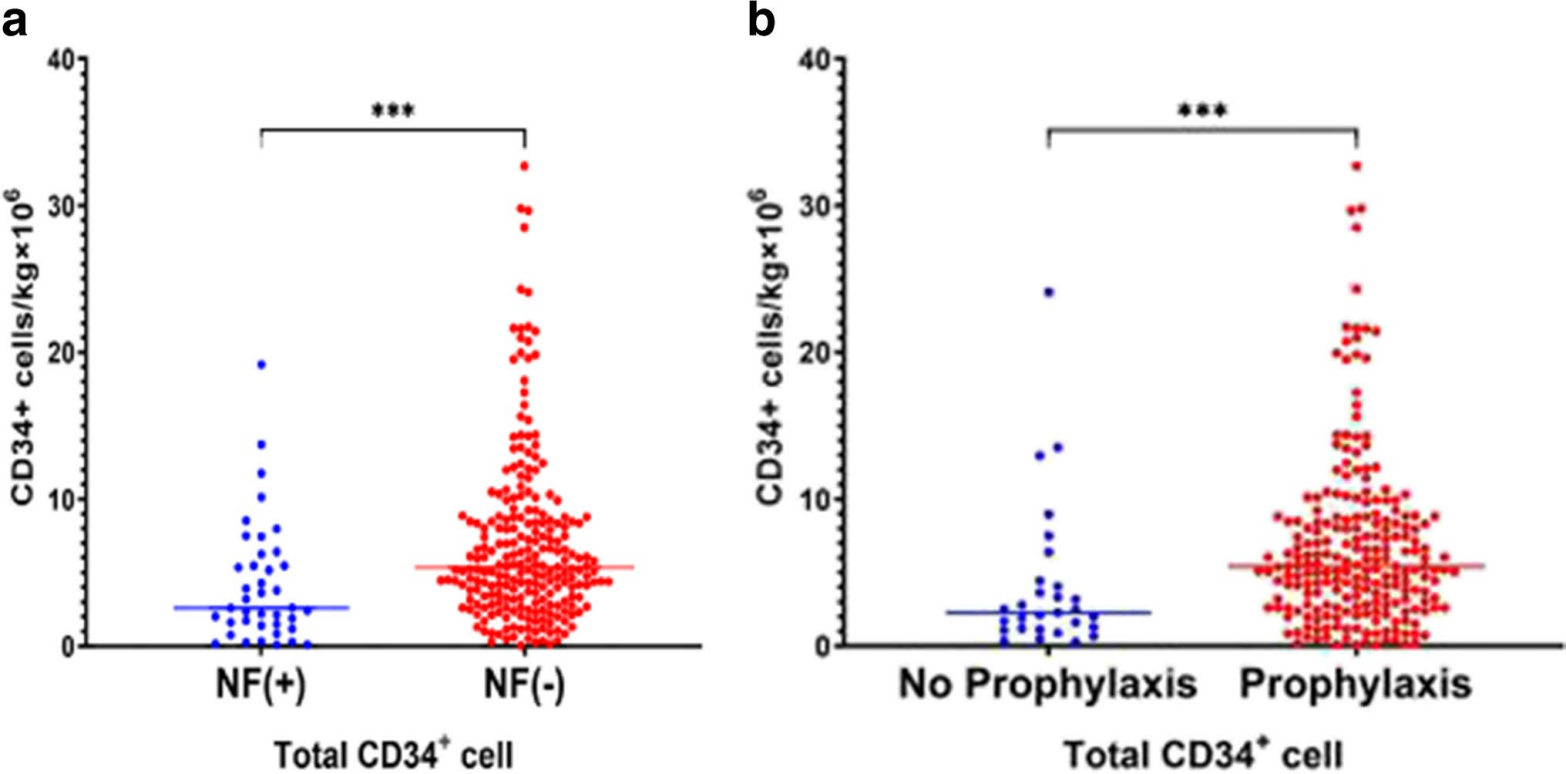
Total CD34 + cell yield in NF ( +) group and NF( −) group (**a**). Total CD34 + cell yield in prophylaxis group and no prophylaxis group (**b**)

Twelve patients (27. 2%) in the NF( +) group did not proceed to ASCT after one mobilization, including four patients who did not collect stem cells in the first mobilization due to fever. One of the patients who did not collect due to fever was converted to bone marrow transplantation, and three patients successfully entered transplantation on second mobilization. Seventeen patients (7.3%) in the NF( −) group did not proceed to ASCT after one mobilization, including five patients who were converted to bone marrow transplantation, one patient who changed to a no-transplant treatment, and 11 patients who successfully entered ASCT after second mobilization. Those patients in the NF ( +) group had a lower likelihood of proceeding to transplant after a single mobilization (72.7% versus 92.7%, *p* < 0.001). The total CD34 + cell yield (median, 2.60 versus 5.34 × 10^6^/kg, *P* < 0.001) and day 1 CD34 + cell yield (1.28 versus 2.5 × 10^6^/kg, *P* < 0.001) were lower in the NF( +) group (Fig. 1). Fewer patients in the NF( +) group had more than 2 × 10^6^/kg CD34 + cells (65.0% versus 88.6%, *P* = 0.001), more than 4 × 10^6^/kg CD34 + cells (37.5% versus 68.8%, *P* < 0.001), and more than 5 × 10^6^/kg CD34 + cells (35.0% versus 53.8%, *P* = 0.027). The days between the administration of chemotherapy and the initiation of apheresis were significantly longer for NF( +) patients than for NF( −) patients (median 10.0 versus 9.0 days, *P* < 0.001). The total apheresis time was longer in the NF( +) group (median, 3 versus 2 days, *P* = 0.007) than in the NF( −) group. Neutrophil and platelet engraftment occurred in all transplant recipients. Neutrophil engraftment (median, 11 versus 10 days, *P* = 0.002) and platelet engraftment (median, 13 versus 11 days, *P* = 0.043) were faster in the NF( +) group (Table 2).

**Table 2:** Stem cell mobilization and transplant outcomes in NF ( +) group and NF ( −) group

Variable	NF ( +) (*n* = 40)	NF ( −) (*n* = 234)	*P* value
Total CD34 + cells collected, × 10^6^/kg, median (IQR)	2.60 (1.43, 6.07)	5.34 (3.17, 8.77)	0.000
Day1 CD34 + cells collected, × 10^6^/kg, median (IQR)	1.28 (1.48, 2.34)	2.5 (0.94, 5.03)	0.000
No. of patients who achieved > 2 × 10^6^/kg (*n*, %)	26 (65.00%)	203 (88.74%)	0.001
No. of patients who achieved > 4 × 10^6^/kg (*n*, %)	15 (37.50%)	161 (68.80%)	0.000
No. of patients who achieved > 5 × 10^6^/kg (*n*, %)	14 (35.00%)	126 (53.85%)	0.027
Days between administration of chemotherapy and initiation of apheresis, median(IQR)	10 (9, 11)	9 (8,10)	0.000
Apheresis collections, median (IQR)	3 (2,3)	2 (2,3)	0.007
Infused CD34 + /kg CD34 + cells transfused, median (IQR)	2.34 (1.22,3.64)	3.32 (2.36, 5.16)	0.001
Days to neutrophil engraftment, median (IQR)	11 (10, 12)	10 (9, 11)	0.002
Days to platelets engraftment, median (IQR)	13 (10, 14)	11 (10, 14)	0.043

### Survival of the NF ( +) group and NF( −) group

**Fig. 2:**
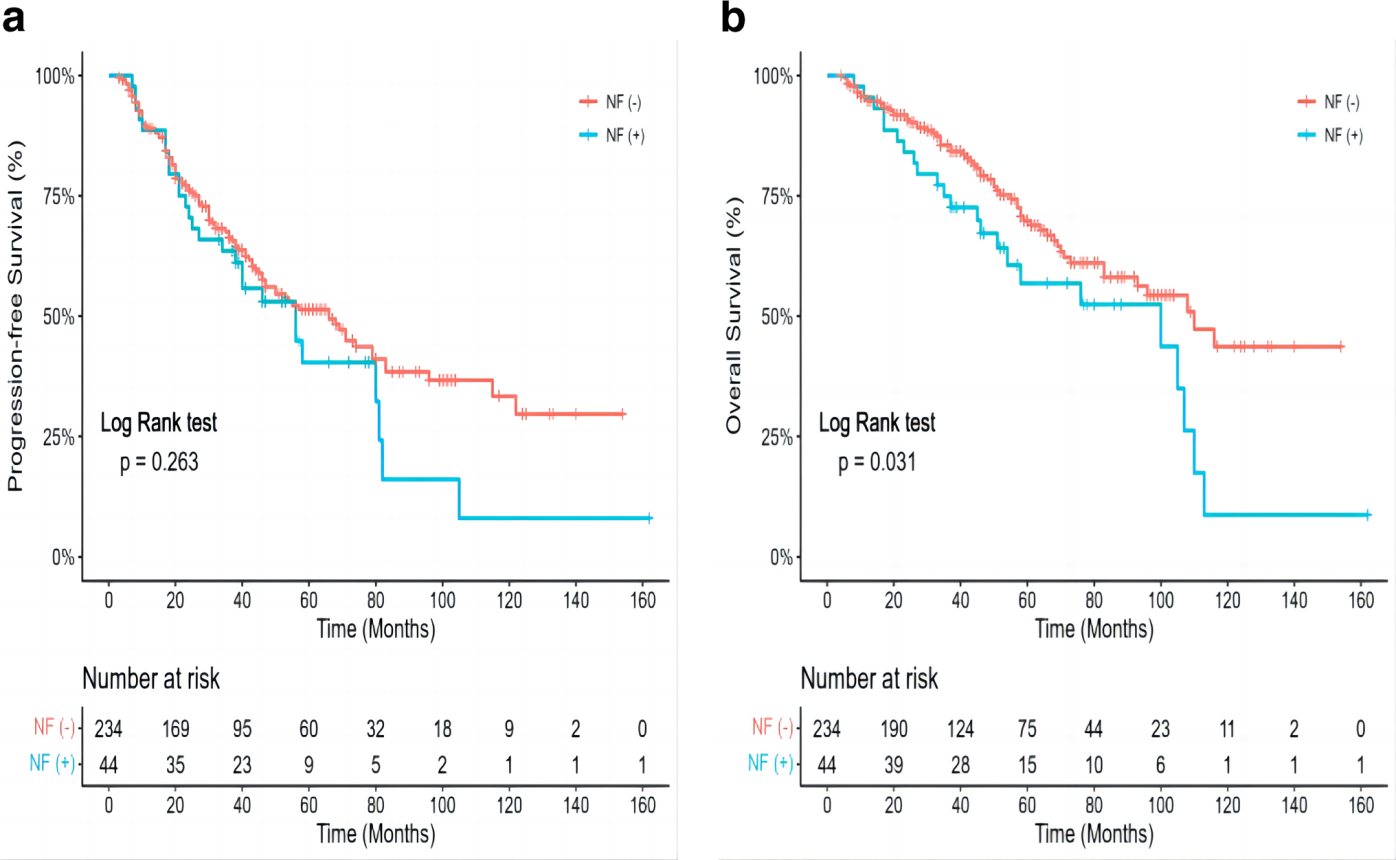
Progression-free survival in NF ( +) group and NF( −) group (**a**). Overall survival in NF ( +) group and NF( −) group (**b**)

For all patients who underwent HD-CY plus G-CSF mobilization, NF ( +) patients had worse OS after mobilization compared to those who did not have NF (median 110.00 ± 11.71 versus 78.00 ± 14.45 months, *P* = 0.011). However, there were no statistically significant differences in PFS between the NF( +) group and the NF( −) group (66.00 ± 8.59 versus 56.00 ± 10.01 months, *P* = 0.249) (Fig. 2). On multivariable analysis, NF was not found to be associated with overall mortality (HR 1.27, 0.706–2.294, *P* = 0.422). Older age at transplant, need for greater than four apheresis results in 2 × 10^6^/kg CD34 + cells, and longer time from diagnosis to mobilization were risk factors for adverse mortality.

### Patient demographics of the prophylaxis group and no prophylaxis group

Thirty patients were mobilized with HD-CY plus G-CSF with prophylactic antibiotics, and 227 patients without routine use of antibiotics were included in our study. Table 3 shows that the prophylaxis group and groups without prophylaxis did not differ significantly in terms of age, sex, ISS stage, number of prior chemotherapy regimens, disease status before mobilization, prior exposure to lenalidomide or alkylating agents, or courses of prior radiation therapy (Table 3).

**Table 3:** Baseline characteristics at diagnosis of study population of prophylaxis group and no prophylaxis group

Variable	Prophylaxis (*n* = 227)	No Prophylaxis (*n* = 30)	*P* value
Mean age, year, (± SD)	52.08 ± 9.72	52.13 ± 8.80	0.973
Male, *n* (%)	140 (62.2%)	20 (66.7%)	0.904
Time from diagnosis to mobilization (mo), median (IQR)	3.00(2.00, 6.25)	5.00(4.00, 6.00)	0.006
ISS, *n* (%)			0.224
I	76(33.8%)	6(20%)	
II	99(44%)	14(46.7%)	
III	50(22.2%)	10(33.3%)	
Prior radiation therapy, *n* (%)	2(0.9%)	0	1.000
Number of prior chemotherapy regimens, *n* (%)			0.473
1	174(77.3%)	26 (86.7%)	
2	40(17.8%)	2 (6.7%)	
≥ 3	11(4.9%)	2(6.7%)	
Disease status before mobilization, *n* (%)			0.132
CR	83 (36.9%)	8 (26.7%)	
VGPR	112(49.8%）	13 (43.3%)	
PR	25 (11.1%)	8 (26.7%)	
SD	4 (1.8%)	1 (3.3%)	
PD	1 (0.05%)	0	
Previous lenalidomide exposure, *n* (%)			0.08
None	190(84.4%)	30 (100%)	
≤ 4 cycles	23(10.2%)	0 (0%)	
≥ 5 cycles	12(5.3%)	0 (0%)	
Prior exposure to alkylating agent, *n* (%)			0.286
Yes	7 (3.1%)	2 (6.7%)	
No	218(96.9%)	28 (93.3%)	
Percent bone marrow PCs at diagnosis, median (IQR)	31.25%(9.75%, 47.75%)	23%(12%, 41%)	0.657
Hemoglobin at diagnosis (g/L), mean ± SD	96.6 ± 22.79	100.01 ± 25.07	0.481
Serum creatinine at diagnosis (umol/L), median (IQR)	89.50(69.5, 123.25)	85.00(64.00, 112.5)	0.317
Serum albumin at diagnosis (g/L), mean ± SD	35.08 ± 6.97	37.44 ± 8.47	0.09
Serum calcium at diagnosis (mmol/L), median (IQR)	2.39(2.26,2.50)	2.36(2.20, 2.50)	0.409
LDH at diagnosis (U/L), median (IQR)	166.00(129.00, 254.40)	153.00(126.00, 206.75)	0.498
Type of myeloma, *n* (%)			0.70
IgG	118 (52.4%)	17 (56.7%)	
IgA	50 (22.2%)	7 (23.3%)	
IgD	10 (4.4%)	0(0.0%)	
Light chains only	47 (20.9%)	6 (20.2%)	

*ISS* international staging system, *CR* complete response, *VGPR* very good partial response, *PR* partial response, *SD* stable disease, *PD* progressive disease, *LDH* lactate dehydrogenase

### Stem cell mobilization and transplant outcome of the prophylaxis group and no prophylaxis group

NF occurred in 8/30 (26.7%) nonprophylaxis patients but only 21/227 (9.3%) prophylaxis patients (*P* = 0.01). One patient in the prophylaxis group and three patients in the nonprophylaxis group did not collect stem cells in the first mobilization due to fever. The total CD34 + cell yield (median 5.44 versus 2.18 × 10^6^/kg, *P* < 0.001) and day 1 CD34 + cell yield (2.30 versus 1.95 × 10^6^/kg, *P* < 0.001) were higher in the prophylaxed patients. More patients in the prophylaxis group had more than 2 × 10^6^ CD34 + cells/kg (83.9% versus 53.3%, *P* < 0.01), more than 4 × 10^6^ CD34 + cells/kg (66.4% versus 30%, *P* < 0.01), and more than 5 × 10^6^ CD34 + cells/kg (54.7% versus 23.3%, *P* < 0.01). The days of hospitalization and the days between administration of chemotherapy and initiation of apheresis were significantly longer in nonprophylactic patients than in prophylactic patients (median 12.0 days versus 11.0 days, *P* < 0.01; and median 10.0 days versus 9.0 days, *P* < 0.01, respectively). No significant differences were observed between the prophylactic and nonprophylactic groups in the duration of neutropenia and the median duration of fever. The antibiotic prophylaxis patients had a lower hospitalization cost of mobilization ($ median 3108.02 versus 3702.39, *p* = 0.012) compared to the nonprophylaxed groups. Neutrophil engraftment and platelet engraftment were faster in the prophylaxis group (12 versus 10 days, *P* = 0.000; and 14 versus 12 days, *P* = 0.006, respectively) (Table 4). In addition, in univariable and multivariable analyses, lack of antibiotic prophylaxis (OR 3.951, CI 1.473–10.593, *p* = 0.006) and ISS stage III at diagnosis (OR 1.818, CI 1.045–3.164, *p* = 0.034) were risk factors for NF (Table 5).

**Table 4:** Stem cell mobilization and transplant outcomes in prophylaxis group and no prophylaxis group

Variable	No Prophylaxis (*n* = 30)	Prophylaxis(*n* = 227)	*P* value
Neutropenic fever ( +)	8/30 (26.7%)	21/227 (9.3%)	0.01
Infection ( +)	10/30 (33%)	33/227 (14.6%)	0.017
Total CD34 + cells collected, × 10^6^/kg median (IQR)	2.18 (1.11, 4.58)	5.44 (2.79, 8.77)	0.000
Day 1 CD34 + cells collected, × 10^6^/kg median (IQR)	1.88 (0.87, 3.84)	2.30 (0.83, 4.79)	0.000
No. of patients who achieved > 2 × 10^6^/kg (*n*, %)	16 (53.3%)	187 (83.9%)	0.000
No. of patients who achieved > 4 × 10^6^/kg (*n*, %)	9 (30%)	148 (66.4%)	0.000
No. of patients who achieved > 5 × 10^6^/kg (n, %)	7 (23.3%)	122 (54.7%)	0.001
No. of patients who achieved > 6 × 10^6^/kg (*n*, %)	6 (20%)	99 (44.4%)	0.017
Days between administration of chemotherapy and initiation of leukapheresis median (IQR)	10 (9, 11.5)	9 (9, 10)	0.006
Days of hospitalization median (IQR)	12 (11, 14)	11 (10, 12)	0.037
Cost of PBSC mobilization, dollar median (IQR)	3108.02 (2869.29, 3917.71)	3702.39 (2551.56, 3921.98)	0.242
CD34 + cells transfused, × 10^6^/kg median (IQR)	2.1 (1.09, 3.48)	2.1 (1.09, 3.48)	0.001
Days to neutrophil engraftment, median (IQR)	12 (11, 14)	10 (9, 11)	0.000
Days to platelets engraftment, median (IQR)	14 (11, 19)	12 (10, 13)	0.006

**Table 5:** Univariate and multivariate analysis of parameters affecting the risk of NF

	Univariate analyses			Multivariate analyses		
Parameters	*R*	OR(95%CI)	*P* value	*R*	OR(95%CI)	*P* value
Age of the patients	0.000	1.0 (0.958–1.045)	0.985	0.000	1.00 (0.975–1.046)	0.986
ISS stage(II/III) at diagnosis	0.615	1.850 (1.081–3.164)	**0.025**	0.598	1.818(1.045–3.164)	**0.034**
Prior exposure to lenalidomide greater than 4 cycles	0. 102	1.108 (0.526–2.331)	0.787	0.255	1.291(0.603–2.766)	0.603
Antibiotic prophylaxis	1.267	3.550 (1.407–8.956)	**0.007**	1.374	3.951(1.473–10.593)	**0.006**
Disease status before stem cell mobilization	0.491	1.634 (0.469–5.685)	0.441	0.827	2.286(0.617, 8.471)	0.216

*ISS* international staging system, Stepwise Cox proportional hazards analysis with a variable entry criterion of *p*≤0.10, and a variable retention criterion of *p*≤0.05 was used to identify multivariable risk factors

Most patients with NF did not have a documented source of infection. *Staphylococcus aureus* was cultured from the skin and soft tissue secretions of the right ankle of one patient in the antibiotic prophylaxis group. There were no patients with positive blood cultures in either group. One patient in the antibiotic prophylaxis group had herpes zoster virus infection. No invasive fungal infections or carbapenem-resistant Enterobacteriaceae bacteria (CRE) infections were diagnosed in either group.

## Discussion

This study shows that NF after Cy plus G-CSF mobilization in MM patients is associated with poor stem cell collection. NF patients had a lower likelihood of proceeding to ASCT after one mobilization, and lower CD34 + cell dose collection with a need for more days of apheresis to achieve the target of collection greater than 2 × 10^6^ CD34 + cells/kg stem cells.

The results were similar to the present study of Jack Khouri, which included 593 adult patients with lymphoma who underwent PBSC mobilization with etoposide and G-CSF [10]. Putkonen et al. analyzed 124 MM patients and compared those who failed to mobilize enough CD34 + cells (peak blood CD34 cell count < 20 × 10^6^) (*n* = 20) with those with successful mobilization (*n* = 104). They found that the occurrence of sepsis during mobilization was a significant predictive factor for mobilization failure in univariate (*p* = 0.001) and multivariate analyses (*p* = 0.040) [15]. Johnson et al. found that infection at the time of PBSC harvest adversely affects PBSC yield. Their study included 44 PBSC collections in 23 patients with MM mobilization with HD-Cy plus G-CSF. The study showed that 29 leukaphereses were performed in patients without infection in whom an adequate PBSC yield was obtained in 21 (72%) cases. In 15 procedures performed in patients with infection, the yield was satisfactory in six (40%) (*P* = 0.036) [16]. Topcuoglu et al. performed a retrospective analysis in 84 patients with multiple myeloma and Hodgkin or non-Hodgkin lymphoma, and most of the patients received cyclophosphamide alone or combined with etoposide followed by G-CSF mobilization. The total collected CD34 + cell doses (median, 10.5 × 10^6^/kg versus 9.8 × 10^6^/kg, *P* = 0.9) were similar in patients regardless of NF [11].The different results may be due to the small sample size and the greater heterogeneity of the included population of this study.

The inflammatory cytokines released by infection with their negative effect on the capacity of hematopoietic stem cell (HSC) self-renewal of stem cells may have a role in this poor mobilization. A previous study found that an IL-6 level above the cutoff of 32 pg/ml implied a higher risk of poor mobilization [17]. Studies have shown that TLR4 activation by LPS infection and IFN-γ, which is one of the critical cytokines controlling inflammation, can impair the self-renewal capacity of HSCs [18]. Exposure to IL-1 decreases the self-renewal ability of HSCs [19]. Tumor necrosis factor alpha (TNFa) is a well-established proinflammatory cytokine whose stimulation can also impair the function of HSCs. Pronk et al. reported that targeted deletion of tumor necrosis factor receptor (TNFR) increased the capacity of HSC self-renewal [20]. In another recent study, Yamashita et al. demonstrated that long-term TNF exposure resulted in the inactivation of NF-kB and induced HSCs to undergo necroptosis [21]. Inflammatory cytokines can also affect HSCs directly by promoting proliferation and inducing DNA damage [22].

Because the costs of mobilization failure and second mobilization attempts are considerable, it is important to maximize the likelihood that patients will mobilize adequately on their first attempt. In addition, NF may lead to mobilization failure and missed opportunities for stem cell collection, and many clinicians are beginning to use antibiotic prophylaxis. The purpose of using prophylactic antibiotics is to decrease infectious episodes, mobilization failure rates, and costs. There are no phase II or randomized studies that have evaluated the safety and efficacy of prophylactic antibiotics among patients receiving HD-Cy plus G-CSF mobilization. In response to this, our study showed that carbapenem antibiotic prophylaxis during mobilization was instituted with a striking reduction in the rate for neutropenic fever, from 26.7 to 9.3%. The study of Avery et al. showed that the incidence of NF associated with etoposide mobilization can be reduced by vancomycin i.v., cefepime i.v., clarithromycin p.o., and ciprofloxacin p.o. or vancomycin i.v., clarithromycin p.o., and ciprofloxacin p.o. from 68 to 26% [12].The study of Yang showed that the addition of antibiotics (ciprofloxacin) reduced the rate of neutropenic fever from 1 to 6%. However, the difference between groups was not statistically significant [13]. The antibiotic prophylaxis regimen in our study is different from the two studies above. A single antibiotic prophylaxis regimen in our study can significantly reduce the rate of neutropenic fever. In addition, several problems have emerged from the use of quinolone prophylaxis. A major concern is that quinolone prophylaxis may increase the risk for developing infections with resistant bacteria, as reported in several studies carried out in patients treated with conventional chemotherapy, which showed that patients receiving fluoroquinolone prophylaxis rapidly become colonized with quinolone-resistant *E. coli* and coagulase-negative staphylococci [23–27]. Carbapenems have a wide spectrum of activity, including gram-positive bacteria such as *Streptococci viridans*, and gram-negative bacteria including *Pseudomonas aeruginosa* and anaerobic bacteria. This wide spectrum of activity and their low range of toxicity support the use of these antibiotics in PBSC mobilization. In the present study, we evaluated the efficacy of antibiotic prophylaxis with carbapenems starting on the first day with a neutrophils count of 0.5 × 10^9^/L after cyclophosphamide mobilization. The present study shows a significant reduction in febrile episodes.

Our study also showed that antibiotic prophylaxis patients had higher CD34 + cell dose collection, and a higher proportion of patients obtained a collection target greater than 2 × 10^6^ CD34 + cells/kg and shorter hospital stays. Thus, due to antibiotic prophylaxis reducing the incidence of NF and leading to improved collection results. The study of Avery et al. showed that there was no significant difference in the total CD34 + cell dose collection between the antibiotic prophylaxis group and the no antibiotic prophylaxis group in high-dose VP-16 mobilization. Unlike our study, the study included patients with ovarian cancer, prostate cancer, and multiple myeloma [12]. The study of Yang et al. showed that more CD34 + cells were collected in the antibiotic prophylaxis group than in the no antibiotic prophylaxis group (9.07 versus 9.85, *p* < 0.01) in HD-Cy plus on-demand plerixafor mobilization, but the difference was not clinically significant [24]. This may be because the dose of cyclophosphamide was lower than the dose in our study, and the incidence of NF in this study was lower than that in our study, leading to no significant difference in the influence of NF on stem cell collection. This broad-spectrum regimen in our study raises some theoretical concerns, such as the potential to select for resistant bacterial organisms or fungi. In fact, we have not observed an increased risk for gram-positive infections among patients receiving prophylaxis with carbapenem, nor an increased risk of fungal infections as suggested by culture analysis for patients whether they received prophylaxis. This could be because the duration of neutrophil recovery and prophylactic antibiotics were quite short. Thus, the broad-spectrum prophylactic regimen utilized here did not appear to have significant adverse effects in terms of selecting resistant organisms.

The economic impact of the procedure is another important aspect regarding the use of prophylaxis with any antibiotics. In the current study, we confirmed that the antibiotic prophylaxis patients had a lower hospitalization cost of mobilization ($ median 3108.02 versus 3702.39, *p* = 0.012) than the unprophylaxed patients. Although the preventive application of antibiotics in the antibiotic prophylaxis group increased the mobilization cost, the total mobilization cost of antibiotic prophylaxis was lower than that in the expense mobilization group. This is mainly due to the higher incidence of febrile episodes among patients in the nonantibiotic prophylaxis group and to the higher percentage of patients in the nonantibiotic prophylaxis group who required a second line of antibiotics. Moreover, the nonantibiotic prophylaxis group had a higher proportion of patients who failed to mobilize due to fever and needed secondary mobilization, which would cost more. In addition, our study also showed that more patients in the prophylaxis group collected more than 2 × 10^6^ CD34 + cells/kg (83.9% versus 53.3%; *P* < 0.01). The remobilization of these patients who did not reach the collection target will also cost more. However, some patients gave up transplantation due to the failure of the first mobilization, and some chose bone marrow transplantation. Due to this, we cannot calculate the remobilization cost of these patients. If the mobilization cost of these patients is calculated, the nonantibiotic prophylaxis group will spend more on secondary mobilization than the antibiotic prophylaxis group.

Although the risk of infection in multiple myeloma patients with neutropenia after chemotherapy mobilization is high, no specific antibiotic prophylaxis guidelines are available for these patients. According to recent guidelines [28, 29], antibacterial prophylaxis is recommended for patients who are at a high risk of infection, including patients likely to develop profound, protracted neutropenia (neutrophils < 0.1/µL for > 7 days) and patients who have neutropenia > 0 and ≤ 7 days with additional risk factors. However, the definition of “additional risk factors” in the guidelines is vague. Although most of the patients in our study may not meet the high-risk criteria for the depth and duration of neutropenia, each of them had at least two of the “additional risk factors” mentioned in the guidelines, including a diagnosis of MM and a dose of cyclophosphamide ≥ 1 g/m^3^. In addition, most infections in patients with myeloma are not related to low neutrophil counts but are due to immune deficiencies, including reduced innate and adaptive immunity, reduced barrier integrity, and the introduction of immunotherapy. Therefore, we think that the patients in our study have a high risk of infection. Given that patients at high risk of NF could also be at increased risk for mobilization failure and that prophylactic use of antibiotics can reduce the rate of NF with acceptable side effects, we recommend that future guidelines classify this group of patients as at high risk for infection and give them antibiotic prophylaxis to prevent NF, improve stem cell mobilization, and collection outcomes.

Indeed, current guidelines recommend fluoroquinolone rather than carbapenems to prevent NF in neutropenic patients. In recent years, we have witnessed an increase in bloodstream infections due to multidrug-resistant organisms (MDROs) in patients with hematologic tumors and neutrophilic deficiency in our country [30, 31]. Broad-spectrum B-lactamase (ESBL) production in *E. coli* is commonly involved, and these strains are usually resistant to fluoroquinolone. Fluoroquinolone prophylaxis is unlikely to be effective when the level of resistance within *E. coli* isolates from internal medicine patients at an institution approaches 20% [32]. However, the routine use of carbapenems as prophylaxis in MM patients with neutropenia after chemotherapy mobilization may initially seem inconsistent with antimicrobial management principles (AMS). But the effective prophylaxis with carbapenems prevents serious infection, thereby reducing subsequent antibiotic use, the patient’s time in the hospital, and even the chance of going to the intensive care unit (ICU). Reducing the length of hospital stay and avoiding ICU visits can help avoid exposure to multidrug-resistant bacteria and thus comply with AMS principles. Because this study was not a clinical trial comparing fluoroquinolone and carbapenem prophylaxis, we cannot draw conclusions as to the effectiveness or selective effect of fluoroquinolone and carbapenem prophylaxis for MDROs. Future RCTs with larger sample sizes are needed to answer this question. Imipenem prophylaxis for patients with multiple myeloma receiving high-dose cyclophosphamide for stem cell mobilization has proven highly effective at our institution. In our study, we did not observe any carbapenem-resistant infections, although the possibility of the emergence of resistant strains should always be considered. This approach could be considered in other centers where the prevalence of carbapenem resistance is similarly low*.* However, antimicrobial stewardship concerns remain.

The limitations of our study include its retrospective nature and lack of randomization. The second limitation is the small number of patients enrolled in the antibiotic prophylaxis group. Therefore, randomized controlled trials with a large sample size are needed to evaluate the efficacy of prophylactic antibiotics for the prevention of neutropenic fever associated with HD-Cy plus G-CSF for PBSC mobilization in the future.

## Conclusion

Patients with NF had a lower likelihood of proceeding to transplant, lower CD34 + cell dose collection, and more apheresis days were required for collection in cyclophosphamide mobilization. The high rate of neutropenic fever seen in the initial patient group was drastically reduced when a broad-spectrum antimicrobial prophylaxis regimen was instituted. This regimen was well tolerated and did not appear to predispose patients to developing infections with more resistant organisms. A regimen of carbapenems appears to be safe and effective in preventing NF associated with HD-Cy plus G-CSF for PBSC mobilization and thus helps to improve PBSC mobilization and collection.

## Data Availability

The processed data required to reproduce these findings cannot be shared at this time as the data also is a part of an ongoing study.
